# Frequency and Nature of Road Traffic Injuries: Data of More than 10,000 Patients from Ha'il, Saudi Arabia

**DOI:** 10.7759/cureus.3830

**Published:** 2019-01-05

**Authors:** Saeed Ahmed, Muzammal Mahmood, Syed Asad Hasan Rizvi, Amna A Siddiqui, Naureen Shahid, Wafa A Akram, Sajila Bano

**Affiliations:** 1 Surgery, Civil Hospital Karachi, Karachi, PAK; 2 Emergency Medicine, King Khalid Hospital, Hail, SAU; 3 Miscellaneous, Civil Hospital Karachi, Karachi, PAK; 4 Surgery, Pakistan Airforce Hospital Masroor Base, Karachi, PAK

**Keywords:** road traffic accidents, fractures, lacerations, amputation, emergency, frequency

## Abstract

Background

Road traffic accidents (RTAs) have become a major issue in today's world. They have caused the loss of more than a million lives in the last decade and are substantially increasing every day. Injuries due to RTAs can cause significant morbidity and mortality. These injuries can be sorted by the type, body region, gender, and age group. We aimed to highlight the frequency and nature of road traffic injuries (RTIs) according to these variables in Ha'il, the Kingdom of Saudi Arabia.

Methods

A retrospective study was conducted in King Khalid Hospital, Ha'il. Data on the cases of RTIs were collected, which presented to the hospital emergency department from January 1, 2016, to December 31, 2017. Data analysis was then performed using IBM Statistical Package for the Social Sciences 17.0 (SPSS, IBM, NY, USA). Frequencies were calculated using descriptive statistics, and graphs were generated.

Results

A total of 10,855 patients with RTIs were encountered at the hospital emergency department. Out of these, 8035 patients were males (74.02%) while 2820 were females (25.97%). Fractures of one or more sites were the most common type of injury, encountered in 5173 patients (47.66%). Lacerations occurred in 3487 patients (32.12%). Crush injuries were encountered in 1190 patients (10.96%) while penetrating injuries occurred in 844 patients (7.78%). Abdominal and pelvic visceral injuries were present in 103 patients (0.95%) while thoracic visceral injuries were present in 39 patients (0.36%). Amputations occurred in 19 patients (0.18%).

Conclusion

Our study highlights the frequency and nature of road traffic injuries that present in the emergency department. As suggested by the high frequency of specific types of injuries, special training should be provided to healthcare professionals to address and treat fractures, head and neck injuries, and serious lacerations effectively.

## Introduction

Road traffic accidents (RTAs) have arisen as an important public health issue, which needs to be addressed immediately. RTAs constitute the majority of the burden of trauma-related hospital admissions worldwide [1]. These RTAs may lead to the injury of one or more body parts, and in severe cases, may also lead to death. Despite continuous efforts to curtail the burden RTAs have imposed on healthcare, the World Health Organization (WHO) reports that 1.25 million individuals lose their lives to these accidents. Around 20-50 million people suffer from non-fatal injuries, including disabilities and trauma, requiring prolonged hospital stays. It is estimated that in the absence of a sustained effort to improve healthcare, road traffic accidents are poised to become the seventh leading cause of death by 2030 [2].

Road traffic injuries (RTIs) may be divided according to the nature or type of injury. The nature or type of RTIs may range from lacerations, penetrating injuries, and crush injuries, to visceral damage, fractures, and amputations. Virtually any body region may be affected by these injuries. According to a study conducted from July 2014 to July 2017, the most frequently injured body region in patients with RTAs included the lower limbs followed by the chest, upper limbs, the head, and the spine [3]. Another study reports intra-abdominal lesions caused by RTAs, in which the larger, solid organs like spleen, liver, and kidneys were reported to be most commonly damaged [4]. This division of the frequency of RTIs according to the nature of the injury and the body region affected may be helpful in a number of ways. It may help to estimate the mortality rate in RTA patients. Literature suggests that patients with severe cerebral and abdominopelvic injuries have a high mortality rate [3]. This may, in turn, provide a better understanding of the severity of individual emergency cases and, therefore, may help the doctors in the emergency department to better triage these cases.

The patients with RTAs may also be divided according to the age group and gender. Since educating people regarding the prevention and safety techniques is required to overcome the burden of RTAs, this division into age group and gender may help to direct the efforts of educating and training the people who are more prone to RTAs than others. Literature suggests that young males are more prone to experience a road traffic accident as compared to the other age groups and genders [1]. Although data from multiple regions around the world have been reported, the literature lacks any data reported from the Ha'il region of the Kingdom of Saudi Arabia (KSA). Through this study, we aimed to report the data from Ha'il region regarding the frequency of RTIs according to the nature or type of RTIs, stratified by gender and age group.

## Materials and methods

A retrospective study was conducted in King Khalid Hospital, Ha'il. After getting ethical approval, data on road traffic injuries were collected from the hospital emergency department. The cases reported from January 1, 2016, to December 31, 2017, were retrieved and grouped according to the nature of RTIs and the body region affected, which were then stratified by age group (<5, 5 to <15, 15 to <25, 25 to <45, 45 to <65, or >65 years) and gender (male or female). Data were then entered into IBM Statistical Package for the Social Sciences 17.0 (SPSS, IBM, NY, USA) and analyzed. Frequencies were calculated using descriptive statistics. Graphs were generated in the end.

## Results

A total of 10,855 patients with road traffic injuries were encountered at the hospital emergency department. Out of these patients, 8035 were males (74.02%) while 2820 were females (25.97%). Among the males, 6037 were Saudi (75.13%) while 1998 were non-Saudi (24.86%). Among the females, 2015 were Saudi (71.45%) while 805 were non-Saudi (28.54%).

When divided by age group, 1063 patients were aged less than five years (9.79%), 2431 belonged to the 5 to <15 years age group (22.40%), 3873 belonged to the 15 to <25 years age group (35.68%), 2420 belonged to the 25 to <45 years age group (22.29%), 978 belonged to the 45 to <65 years age group (9.01%) while 90 were aged more than 65 years (0.83%).

**Figure 1:**
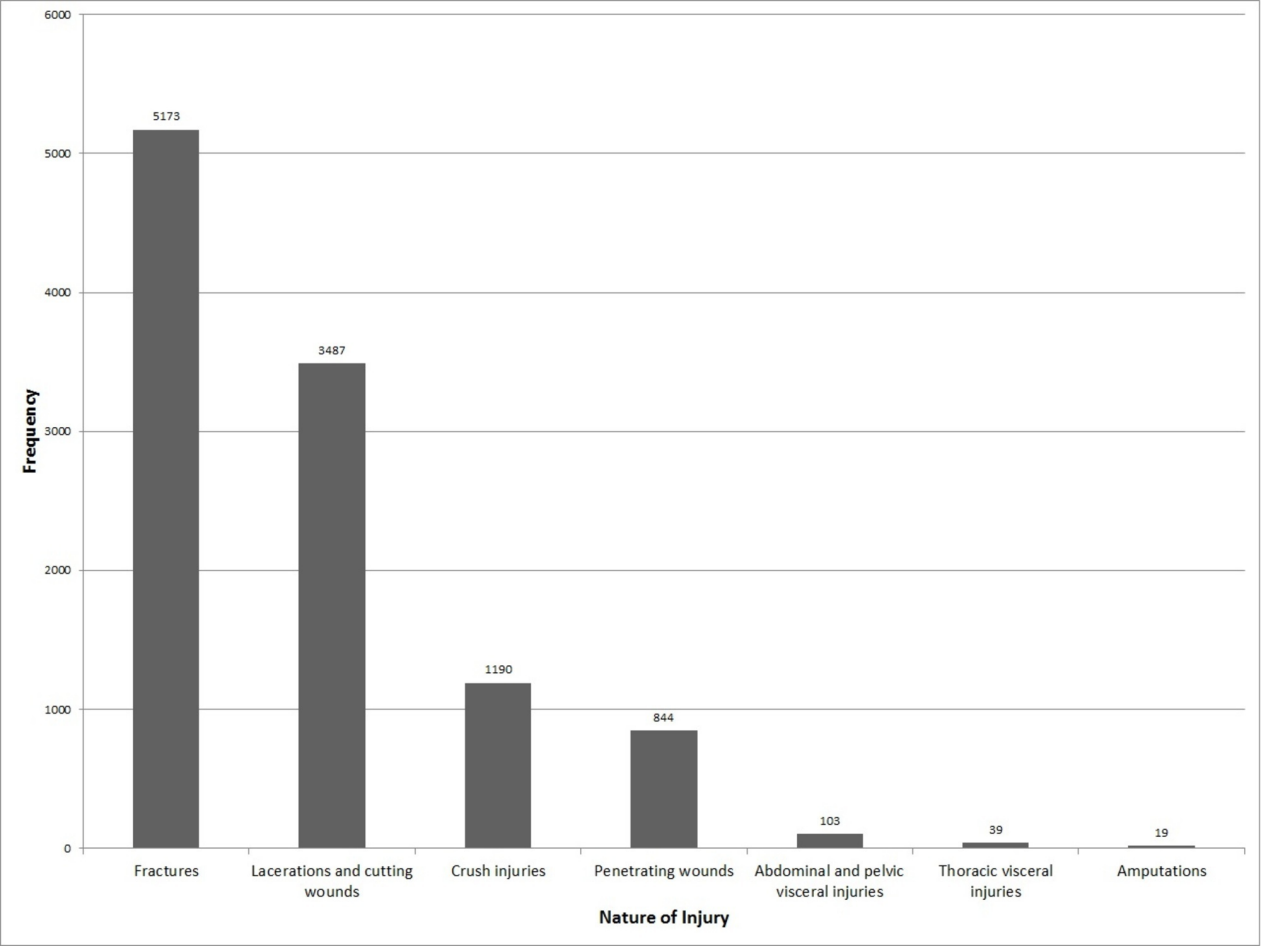
Frequency of road traffic injuries according to their nature

Fractures of one or more sites were the most common type of injury, encountered in 47.66% patients. Lacerations and cutting wounds occurred in 32.12% of the patients. Crush injuries were encountered in 10.96% while penetrating injuries occurred in 7.78%. Abdominal and pelvic visceral injuries were present in 0.95% while thoracic visceral injuries were present in 0.36%. Amputations were encountered in 0.18% of the patients. Figure 1 presents the frequency of these injuries in the form of a column chart.

Table 1 displays the frequency of injuries according to age group. Each type of injury most commonly occurred in the age group of 15 to <25 years.

**Table 1:** Frequency of road traffic injuries according to age groups

Nature of Injury	Age Group						
	<5 years	5-<15 years	15-<25 years	25-<45 years	45-<65 years	>65 years	Total
Fractures	494 (9.55%)	1218 (23.55%)	1936 (37.43%)	1088 (21.03%)	383 (7.40%)	54 (1.04%)	5173 (100%)
Lacerations and cutting wounds	350 (10.04%)	624 (17.90%)	1178 (33.78%)	887 (25.44%)	414 (11.87%)	34 (0.98%)	3487 (100%)
Crush injuries	117 (9.83%)	337 (28.32%)	412 (34.62%)	256 (21.51%)	67 (5.63%)	1 (0.08%)	1190 (100%)
Penetrating injuries	92 (10.90%)	215 (25.47%)	275 (32.58%)	160 (18.96%)	101 (11.97%)	1 (0.12%)	844 (100%)
Abdominal and pelvic visceral injury	9 (8.74%)	23 (22.33%)	49 (47.57%)	22 (21.36%)	0	0	103 (100%)
Thoracic visceral injuries	1 (2.56%)	9 (23.08%)	13 (33.33%)	6 (15.38%)	10 (25.64%)	0	39 (100%)
Amputations	0	5 (26.32%)	10 (52.63%)	1 (5.26%)	3 (15.79%)	0	19 (100%)

Table 2 presents the frequency of injuries according to gender and nationality. Each type of injury most commonly occurred in males, particularly Saudi males.

**Table 2:** Frequency of road traffic injuries according to gender and nationality

Nature of Injury	Gender with Nationality				
	Saudi Male	Saudi Female	Non-Saudi Male	Non-Saudi Female	Total
Fractures	3000 (57.99%)	935 (18.07%)	897 (17.34%)	341 (6.59%)	5173 (100%)
Lacerations and cutting wounds	1868 (53.57%)	716 (20.53%)	619 (17.75%)	284 (8.14%)	3487 (100%)
Crush injuries	554 (46.55%)	232 (19.49%)	300 (25.21%)	104 (8.73%)	1190 (100%)
Penetrating injuries	512 (60.66%)	111 (13.15%)	151 (17.89%)	70 (8.29%)	844 (100%)
Abdominal and pelvic visceral injury	66 (64.07%)	13 (12.62%)	21 (20.38%)	3 (2.91%)	103 (100%)
Thoracic visceral injuries	25 (64.10%)	6 (15.38%)	5 (12.82%)	3 (7.69%)	39 (100%)
Amputations	12 (63.15%)	2 (10.53%)	5 (26.31%)	0	19 (100%)

**Figure 2:**
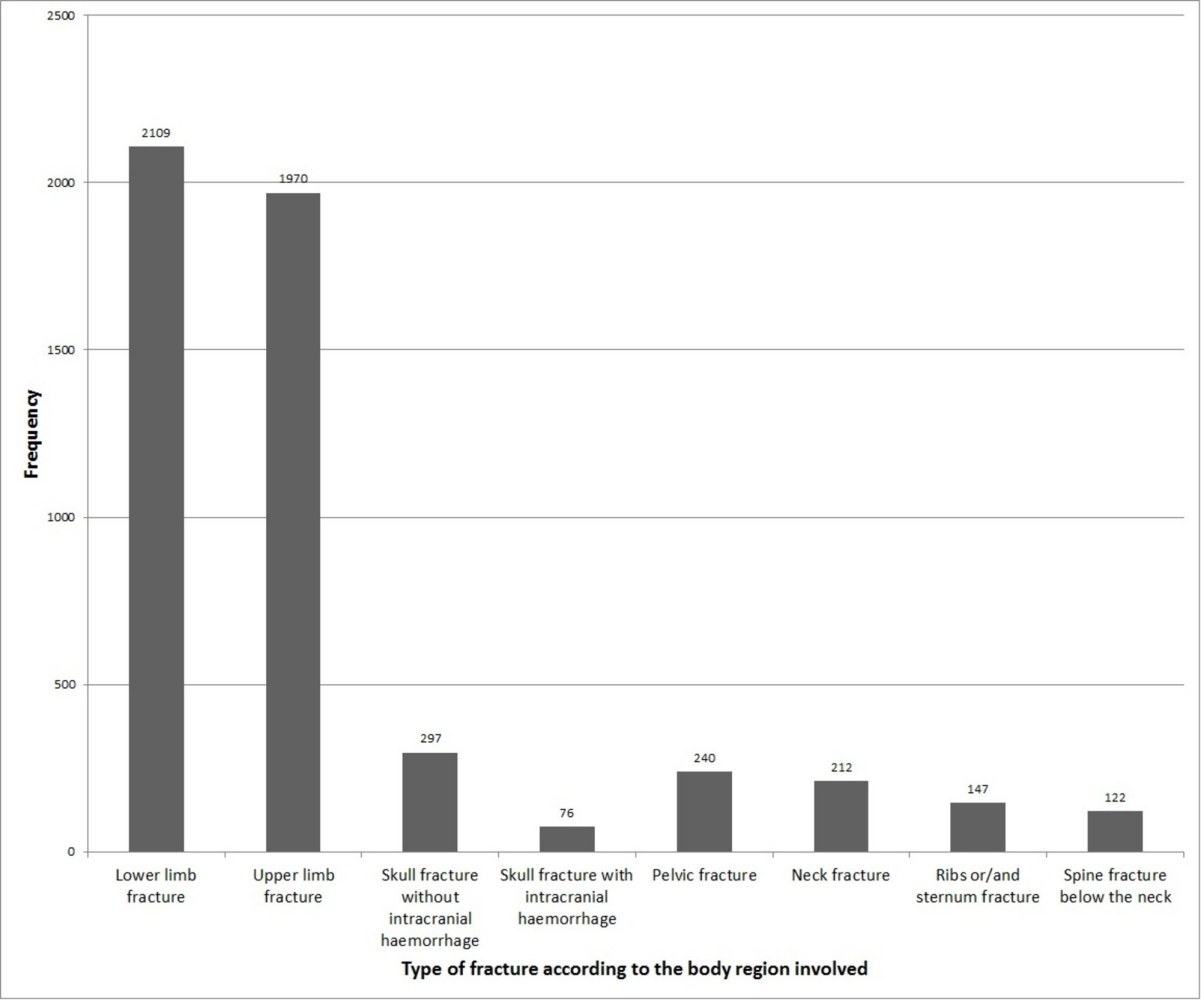
Frequency of fractures according to the involved body region

We further probed the data of patients with fractures to identify the sites of fractures. Among these patients, lower limb fractures were the most common type according to the region involved, occurring in 40.77% patients. Upper limb fractures occurred in 38.08% patients. A skull fracture was present in 7.21%, among which 1.47% patients had an associated intracranial hemorrhage. A fracture of the pelvis occurred in 4.64%. A fracture of the neck was encountered in 4.10%. A fracture of the ribs and sternum was present in 2.84%. A fracture of the spine below the neck was present in 2.36% of the patients. Figure 2 presents the frequency of these fractures in the form of a column chart.

## Discussion

Road traffic accidents and the associated injuries are a common cause of morbidity and mortality in all countries worldwide. In the KSA, fatalities by road traffic accidents account for 4.7% of all mortalities and have increased over the last decade to 27.4 per 100,000 of the population. In contrast to the other developed countries with sound economies, road traffic fatalities do not exceed 1.7% in Australia, the United Kingdom (UK), or the United States of America (USA), and the records indicate an incidence of 10.6 per 100,000 population in the USA and 2.9 per 100,000 in the UK [5]. This may be attributed to strict attention to road safety and rules, with appropriate implementation to ensure primary and secondary prevention regarding RTAs in these countries. Similarly, it has been reported that among other high-income states, Saudi Arabia has a higher number of deaths due to RTAs (accident to death ratio is 32:1 versus 283:1 in the USA) [6]. Injuries due to road traffic accidents are reported to be the most serious in this country with an accident to injury ratio of 8:6 as compared with the international ratio of 8:1 [7]. All of these statistics point to a stark contrast in the incidence rate and the adverse outcomes of RTAs and the resultant injuries and deaths between the KSA and other countries with similar economies and resources. This highlights the urgent need to improve trauma care in the KSA.

According to the morbidity and mortality records in the Ministry of Health (MOH) hospitals, 20% of beds are occupied by RTA victims, and 81% of deaths in the hospitals are due to RTIs [7]. Another study shows that over the past two decades, the KSA has recorded 86,000 deaths and 611,000 injuries in RTAs. With 7% resulting in permanent disabilities [8], 19 killed daily, and four injured every hour in the KSA, RTAs need to be highlighted as a major health hazard in this area [7,9]. The population which is most prone to RTAs are young males, as is demonstrated by our study. A systematic review of studies conducted over two and a half decades revealed the trend of young males having twice the incidence rate for RTI as compared to females [1,10]. In another study [11], an even higher male to female ratio of 4:1 was reported. This discrepancy can be attributed to the driving laws in the KSA, where up until recently, women were not allowed to drive. It can also be insinuated that these figures are a presentation of the prevalent culture in this region with respect to the disproportion of presence between men in women in public areas.

Our study has revealed that in Ha'il, the most common age group to present to the emergency department is from 15 to 25 years of age. The finding that young individuals are more prone to RTAs has been established by several other studies, albeit with variable age groups set for their analyses. According to WHO, people aged between 15 and 44 years account for 48% of global road traffic deaths, and road traffic injuries are the leading cause of death among people aged between 15 and 29 years [2]. In studies conducted in Saudi Arabia, the most common age groups found to be presenting with road accidents and non-fatal associated injuries include 10-19 and 11-30 years [10-11]. A study suggested RTAs to be the country’s main cause of death for 16 to 30-year-old males [6]. Similarly, among 305 individuals presenting at a Saudi trauma center due to road traffic accidents, the median age of young males was 26 years (Interquartile Range, 22-33 years) and that of females was 30 years (Interquartile Range, 28-39) [3]. Another aspect of this information is demonstrated in the fact that in industrialized countries, the gross loss due to accidents is 1 ± 2% of the national income while for the KSA, this loss has been estimated to be between 2.2% and 9% [1,8]. In any nation, the youth of the population is vital to its economy and progress. A higher RTI burden in this age group can have an adverse impact on the well-being of the country.

It is also noteworthy that in a systemic review of all data collected regarding road traffic accidents and admissions in hospitals with RTIs for two decades, a trend was noticed in which young males were found to be affected more than females in all studies with an increased ratio of 4:1 found in past studies and a relatively low ratio of 2:1 in recent studies [1,5,12]. The study also hypothesized the prevalence of accidents among the youth due to a paucity of entertainment avenues present in the country apart from driving. It cited that the use of motor vehicles by young men as an activity of leisure, often in manners that are illegal or reckless, could be attributed to the lack of amusement parks, public outdoor activities and sporting arenas, and events. Lack of control by parents and inadequate training prior to licensure can also be some explanatory factors [1].

According to our study, the most common type of injuries that the patients presented with, after an RTA, were fractures followed by laceration and cutting wounds. In terms of fractures, limbs were the most commonly affected parts, with lower limb fractures more common than upper limb fractures, and head and neck fractures affected prominently less than limbs. This result is comparable to that of a study conducted in Qassam, where data collected within a set spectrum of years revealed head and neck injuries to be the most common [10]. The most common type of presenting injury with an RTA varies over medical literature; in Yemen, the most common type of injury presentation was laceration and superficial wounds, followed by fractures, with internal organ injury being the least common. Lower limbs represented the majority of the total injured body parts followed by the head and the upper limbs [13]. In an Indian study, the most commonly affected area in an RTA was the extremities, followed by the maxillofacial area and then the head and neck. Laceration and cuts were the most common nature of injury in this study, and in terms of fractures, lower extremities were the most commonly affected [14].

## Conclusions

The rate of RTAs in Saudi Arabia stands out among high-income nations with established healthcare systems. To decrease the current burden of RTAs and related injuries, several aspects in the healthcare delivery need to be scrutinized and modified. For the prevention of road crashes, discouraging improper driving, particularly among the young age groups, with strict surveillance and the improvement and implementation of extensive driving education and training is necessary. Furthermore, attention needs to be diverted to the medical care available post-crash and within emergency and trauma departments. As suggested by our data and the medical literature, special training should be provided to healthcare professionals to tackle and treat fractures, head and neck injuries, and serious lacerations effectively.
